# *Bacillus* sp. YC7 from intestines of *Lasioderma serricorne* degrades nicotine due to nicotine dehydrogenase

**DOI:** 10.1186/s13568-023-01593-0

**Published:** 2023-08-21

**Authors:** Ke Zhang, Mingshen Yin, Shengwei Lei, Hongxin Zhang, Xiaoyan Yin, Qiuhong Niu

**Affiliations:** 1 College of Environmental Engineering and Chemistry, Luoyang Institute of Science and Technology, 90 Wangcheng Road, Luoyang, 471023 Henan China; 2 College of Life Science and Agricultural Engineering, Nanyang Normal University, 1638 Wolong Road, Nanyang, 473061 Henan China

**Keywords:** *Lasioderma serricorne*, Endophytic bacteria, Whole genome sequencing, Nicotine degrading enzymes, Gene knockdown, Molecular docking

## Abstract

A large number of nicotine-containing wastes produced during the tobacco manufacturing process are seriously harmful to the environment and human health. The degradation and transformation of nicotine-containing environmental contaminants to harmless substances has become an urgent requirement. *Lasioderma serricorne* can grow and reproduce in nicotine-rich sources, and their intestinal microbiota show promising potential to degrade and utilize nicotine. The purpose of this study is to screen and identify nicotine-degrading bacteria from the intestines of *L. serricorne* and explore their degradation characteristics. A dominant strain, YC7, with significant nicotine degradation capabilities was isolated from the intestines of *L. serricorne.* The strain was identified as *Bacillus* using a polyphasic approach. The test results showed it can produce multiple enzymes that include β-glucosidase, cellulase, proteases, and amylases. The nicotine-degrading bacteria were functionally annotated using databases. Nicotine dehydrogenase (NDH) was found by combining an activity tracking test and protein mass spectrometry analysis. The YC-7 NDH in the pathway was molecularly docked and functionally verified via the gene knockdown method. The binding ability of nicotine to nicotine-degrading enzymes was investigated using molecular docking. A high-efficiency nicotine-degrading bacteria, YC-7, was isolated and screened from tobacco, and the gene functions related to degradation were verified. This investigation provides a new hypothesis for screening nicotine-degrading bacteria and increases our knowledge of potential nicotine-degrading microbial sources.

## Key points

- *Bacillus* sp. YC7 with degrading nicotine ability were isolated from intestines of *Lasioderma serricorne*
- Function of the nicotine dehydrogenase (NDH) in YC7 strain was verified via gene knockdown, qPCR and molecular docking
- The whole genome of YC7 was analyzed to find the characteristics of YC7

## Introduction

Tobacco is an important cash crop that is primarily used to produce tobacco products and pesticide insecticides. Nicotine insecticides have been widely used in agricultural production since the 1980s (Matsuda et al. 2020; Xia et al. 2020), but they produce a large number of toxic wastes during production processing and consumption. Only 5% of these wastes can be absorbed by crops. The remaining wastes spread and diffuse to the atmosphere, soil, water, plants, animals, and the human body, thus seriously polluting the environment (Matsuda et al. 2020) and threatening human health (Xia et al. 2020). Globally, 6.7 Mt of tobacco is produced annually, of which China accounts for the largest share at 39.6%, and the tobacco industry is expected to produce approximately 300,274 t of waste each year (He et al. 2019; Najme et al. 2021).

People pay more attention to health in today’s society, and they are interested in preserving the environment. Hence, the reduction of nicotine content in the environment has become an important topic that requires urgent attention.

In recent years, there has been a growing interest in the development of innovative and sustainable technologies for the degradation of nicotine, a major addictive component of tobacco products. Traditional approaches to nicotine degradation have often involved chemical methods that can be environmentally harmful. As a result, researchers have turned their attention towards alternative and eco-friendly strategies, among which microbial degradation through metabolism has emerged as a promising solution (Tang et al. 2008; Li et al. 2017; Xia et al. 2018; Qiu et al. 2019; Wang et al. 2021).

Tobacco beetles (*Lasioderma serricorne*) have been recognized as a potential source of microbial activity for nicotine degradation (Ren et al. 2022). These tiny insects have shown the ability to metabolize nicotine as part of their natural feeding behavior, and their gut microbiota play a crucial role in this process. The nicotine content in tobacco plays a role in the growth and development of *L.serricorne* (Sakka and Athanassiou 2023); when the content is low, the growth and development of the larvae are good condition. When tobacco leaves were given to tobacco beetles as feed, the growth and development of the larvae remained unaffected even when the nicotine content increased to 4% (Edde 2019). It was concluded that tobacco beetles should be screened for nicotine-degrading bacteria that can degrade nicotine in their bodies.

Within the gut of tobacco beetles, a diverse community of intestinal flora coexists, comprising various microorganisms such as bacteria and fungi. Studies have shown that certain strains of bacteria in the beetle’s gut possess the enzymatic machinery to break down nicotine into less harmful compounds (Huang et al. 2020). This intricate microbial nicotine degradation pathway has caught the attention of researchers as a novel and biologically inspired approach to combat nicotine pollution.

Metabolism, as a novel and alternative technology for nicotine degradation, has garnered significant attention as a new and trending topic. The mechanism underlying microbial nicotine degradation involves a series of enzymatic reactions that transform nicotine into simpler metabolites (Wu et al. 2014; Qiu et al. 2016a; Liu et al. 2020; Shang et al. 2021a, 2021b). One of the primary pathways identified is the stepwise conversion of nicotine to nornicotine, followed by the breakdown of nornicotine into even less toxic compounds (Hu et al. 2019). This degradation process demonstrates the potential to mitigate the environmental impact of nicotine waste and offers a sustainable alternative for its removal.

With the increasing recognition of the adverse effects of nicotine on human health and the environment, the exploration of microbial degradation pathways, especially in tobacco beetles’ intestinal flora, has become a new and exciting research focus (Huang et al. 2020; Mu et al. 2020; Xia et al. 2020; Yu et al. 2021; Zhang et al. 2022). Harnessing the natural biodegradation capabilities of these microorganisms holds the promise of offering a greener and more efficient approach to tackle nicotine pollution.

In this study, we aim to delve deeper into the microbial nicotine degradation pathway within the gut of tobacco beetles, exploring the enzymatic mechanisms involved, and evaluating its potential as a viable technology for nicotine waste remediation. Understanding these processes will not only shed light on the ecological significance of microbial degradation but also pave the way for developing innovative strategies to address nicotine pollution and promote environmental sustainability. In detail, we select the tobacco beetle as the research object. We isolated and screened the nicotine-degrading bacteria in the tobacco beetle and conducted morphology and 16S rRNA sequence analyses. We then conducted a nicotine degradation activity test to study the degradation characteristics of the strain. We determined the key genes and metabolic pathways of nicotine degradation after a whole-genome sequencing analysis, and we predicted the structure and function information of the key genes in nicotine degradation using molecular docking. We then identified the key genes related to nicotine degradation by molecular docking. Finally, a functional identification of the key degradation genes was performed, which in turn provides a theoretical foundation for nicotine pollution treatment.

## Material and methods

### Isolation and differentiation of bacteria and *L. serricorne* grinding

*Lasioderma serricorne* was obtained from Prof. JiaQin Xi of the Zhengzhou Tobacco Research Institute. After rinsing with sterile water and then disinfecting with 75% ethanol on a super-clean bench, the *L. serricorne* was dissected. The mouthparts and gut were collected and ground into a homogenate, a gradient dilution was performed, and 20 μL of each gradient was evenly coated on tobacco plates and incubated at a constant temperature of 37 °C for 12 h. Colonies with different sizes, shapes, and colors were selected for purification (Shen et al. 2021). The growth conditions of the strains were compared to determine the best formulation of the tobacco medium and to re-screen the nicotine-degrading bacteria (Han et al. 2021).

### Identification of physiological and biochemical factors

The strains were subjected to Gram staining, spore staining, a potassium hydroxide (KOH) pulling assay, a β-glucosidase activity assay, a cellulase activity assay, a protease activity assay, and an amylase activity assay.

KOH pulling assay was carried out as follows: Take 20 μL of 4% KOH solution and drop it onto a glass slide. Pick an appropriate amount of bacterial strain and mix it thoroughly with the KOH solution. Every 5 s, use an inoculation loop to lift and pull the mixture to observe if it forms strings.

β-Glucosidase activity assay was performed as follows: Employ a quercetin plate to test the bacterial strain’s β-glucosidase activity. After activating the strain, spot a single colony in the center of the quercetin plate. If the plate turns black after 48 h, it indicates the strain’s production of β-glucosidase.

Cellulase Activity Assay: After activating the strain, spot a single colony on a Congo red plate and incubate for 48 h. Decolorize the plate using a freshly prepared 5 mol/L NaCl solution. The presence of a transparent halo indicates cellulase production by the strain.

Protease Activity Determination: Streak the selected nicotine-degrading bacterial strain on an LB agar plate and incubate it at a constant temperature of 37 °C for 12 h. Observe the single colony, then spot it on a skim milk plate. After incubating for 48 h, the appearance of a transparent halo confirms the strain’s protease production.

Amylase Activity Assay: After activating the strain, spot a single colony in the center of a starch plate. Place the plate in a 37 °C incubator for 48 h and then test for amylase activity by applying iodine solution. The presence of a transparent halo indicates the strain’s ability to produce amylase.

### Taxonomic identification of the strains and construction of the phylogenetic tree

The morphological properties of the obtained highly active strains was observed by lightmicroscopy (Olympus microscope BH-2) and Hitachi H800 transmission electron microscopy. The total bacterial DNA was extracted using alkaline lysis, and the primers and reaction system cited referenced a study to amplify the 16S rRNA gene of the isolated strain. The bacterial strain was sent to Biotech Bioengineering (Shanghai) Co. for sequencing. The obtained sequences were entered into the Ezbiocloud database, and the sequences were subjected to a genetic evolutionary tree construction.

### Degraded nicotine characteristic experiments

Tobacco leaves with 0.96% and 9.6% nicotine were kindly provided by Prof. Jiaqin Xi from the Zhengzhou Tobacco Research Institute. The nicotine was diluted with 0.05 mol/L HCl in different gradients, and OD_259_ was measured, controlling the absorbance values in the range of 0.2–0.8, and the control was 0.05 mol/L HCl. The standard curve was constructed with the horizontal coordinate being the nicotine concentration and the vertical coordinate being the absorbance value. In detail, fermentation solution of the strain to degrade nicotine activity was detected based on the literature (Tang et al. 2008): A single colony of the strain was collected in 5 mL of the nicotine medium (first with a trace elements solution consisting of 0.4 g of MnSO_4_-7H_2_O, 0.2 g of FeSO_4_-7H_2_O, 0.2 g of CaCl_2_-2H_2_O, fixed to 100 mL with 0.1 mol/L HCl). This was incubated at 37 °C and 220 rpm for 12 h as the seed solution. This then received 50 mL of the following solution at a ratio of 1:10 (13.3 g of K_2_HPO_4_, 4.0 g of KH_2_PO_4_, 1.0 g of yeast powder, and 10 mL of the trace element solution. This had ddH_2_O added to 1000 mL at pH 7.0. This was then sterilized at 121 °C for 20 min, and a certain amount of nicotine was added (0.22 μm membrane filtration). This was centrifuged at 8000 rpm for 10 min for different time periods. The absorbance values were measured by OD_259_ with an enzyme marker, and the repercussed nicotine medium without the strain was used as a control for 10 min. The values were entered into the standard curve to calculate the remaining nicotine concentration, and the nicotine degradation rate was calculated.

A total of 1 g of tobacco powder was weighed and inserted into a conical flask, 2 mL of the bacterial suspension (OD = 0.5) was added to the experimental group, and an equal amount of phosphate buffer solution (PBS) was added to the control group. This was incubated for five days at 37 °C, the tobacco leaves were removed, dried by oven at 64 °C, and the nicotine content was determined using hydrochloric acid extraction and the decolorization method. The nicotine degradation rate was then calculated as the formula: Nicotine degradation rate (%) = (Initial nicotine content—Nicotine content in fermentation broth) / Initial nicotine content × 100%

### Test of the extracellular crude enzyme solution for the nicotine degradation activity

The extracellular crude enzyme solution was extracted using ammonium sulfate graded precipitation, dialysis, concentration, and filtration of the fermentation broth at 37 °C for 48 h. The crude enzyme solution was detected by SDS-PAGE electrophoresis, and finally the crude enzyme solution concentration was detected using the bicinchoninic acid (BCA) method. A total of 100 μL of the crude enzyme solution and 100 μL of the nicotine dilution solution (1 mol/L) were added to each well of a 96-well plate in a 1:1 ratio, and PBS was used as the control. The reaction was performed at 37 °C. After 30 min, the nicotine degradation enzyme activity was calculated by comparing with the control group and detecting OD_259_ using an enzyme marker.

A total of 1 g of tobacco end was weighed and placed in a conical flask. A total of 2 mL of the crude enzyme solution (OD = 0.5) was added to the experimental group, and the same amount of PBS was added to the control group. Both groups were incubated at 37 °C for 5 d. The tobacco leaves were removed and dried in an oven at 64 °C, and the nicotine content was determined by hydrochloric acid extraction and the decolorization method. The nicotine degradation rate was then calculated.

### Whole-genome sequencing analysis

Single colonies of the strain were selected and inoculated into 100 mL of the liquid Luria–Bertani (LB) medium. This was incubated at 37 °C in a shaker until the logarithmic growth period and then centrifuged and collected. This was then sent to the Wuhan Bena Technology Service Co. for genome extraction, bacterial library construction, and bioinformatics analysis (Mu et al. 2020; Wang et al. 2019a). We predicted the protein-coding genes of the bacterium by leveraging information from Swiss-Prot (http://web.expasy.org/docs/swiss-prot_guideline.html) and using GeneMarkS software (http://topaz.gatech.edu/). Gene functions were obtained from the Gene Ontology database (GO, http://www.geneontology.org/) through IPRscan, which classifies functions into three categories: cellular component (CC), molecular function (MF), and biological process (BP). Furthermore, we predicted genes involved in signaling pathways by conducting BLAST searches, and we determined the functions of gene-encoded proteins by aligning them with the Cluster of Orthologous Groups of proteins (COG, https://www.ncbi.nlm.nih.gov/COG/) database. The COG database is structured based on the evolutionary relationships of the whole-genome-encoded protein system and is commonly used for protein function deduction. Additionally, we annotated transporters using the Transporter Classification Database (http://www.tcdb.org/) through the BLAST software.

### Construction of the knockdown mutant strain of the nicotine dehydrogenase

The pBD1 recombinant plasmid previously constructed was used to construct a knock down mutant strain of nicotine dehydrogenase (*ndh*) (Niu et al. 2015; Tao et al. 2021). When designing the sgRNA, we searched for the 5′- NGG-3′ sequence on the target gene template chain to complement and bind with the sticky end generated by the Sap I cleavage of the sgRNA expression box. At the 5′ starting end of the YC7 *ndh* gene at the distance from the starting codon, the position of GTG 113 nt contained a non-specific paired binding region of NGG, and a pair of sgRNA1 primers oligo1 (TGTCCTGCGGCAAGGGCATAATA) and oligo2 (AACTATTATGCCCTTGCCGCAGG) targeting *ndh* were designed. The pair of primers were annealed and connected with the pBD1 plasmid to obtain the target plasmid, pBD1-bace16.

After annealing the primers at 95 °C and extending, the primers were ligated into the plasmid digested with SapI to construct the knockdown plasmid pBD1-ndh. The plasmid was transformed into YC7 competent cells by electroporation according to standard protocols. The knockdown mutant strain, KYC7, was obtained after verification. The degrading nicotine capabilities of the mutant strain were measured to test the degradation function of NDH.

### Molecular docking and RT-qPCR functional verification

Based on the results of whole-genome sequencing analysis and KEGG annotation, YC7 was found to possess a key enzyme, NDH, encoded by the gene *ndh*, involved in the initial step of the pyridine pathway for nicotine degradation. Molecular docking was performed with the YC7 NDH as the receptor and nicotine as the ligand, and bioinformatics tools were used to predict the structural and functional information of the key enzyme for nicotine degradation (Linani et al. 2022; Pinzi and Rastelli 2019; Stanzione et al. 2021).

YC7 was induced with 1 g/L nicotine and 0.8% tobacco extract for 24 h. The expression of the YC7 *ndh* gene was then detected using real-time quantitative PCR to see whether the gene appeared to be up-regulated to further determine the gene function.

## Results

### Isolation and screening results of nicotine-degrading bacteria

**Fig. 1:**
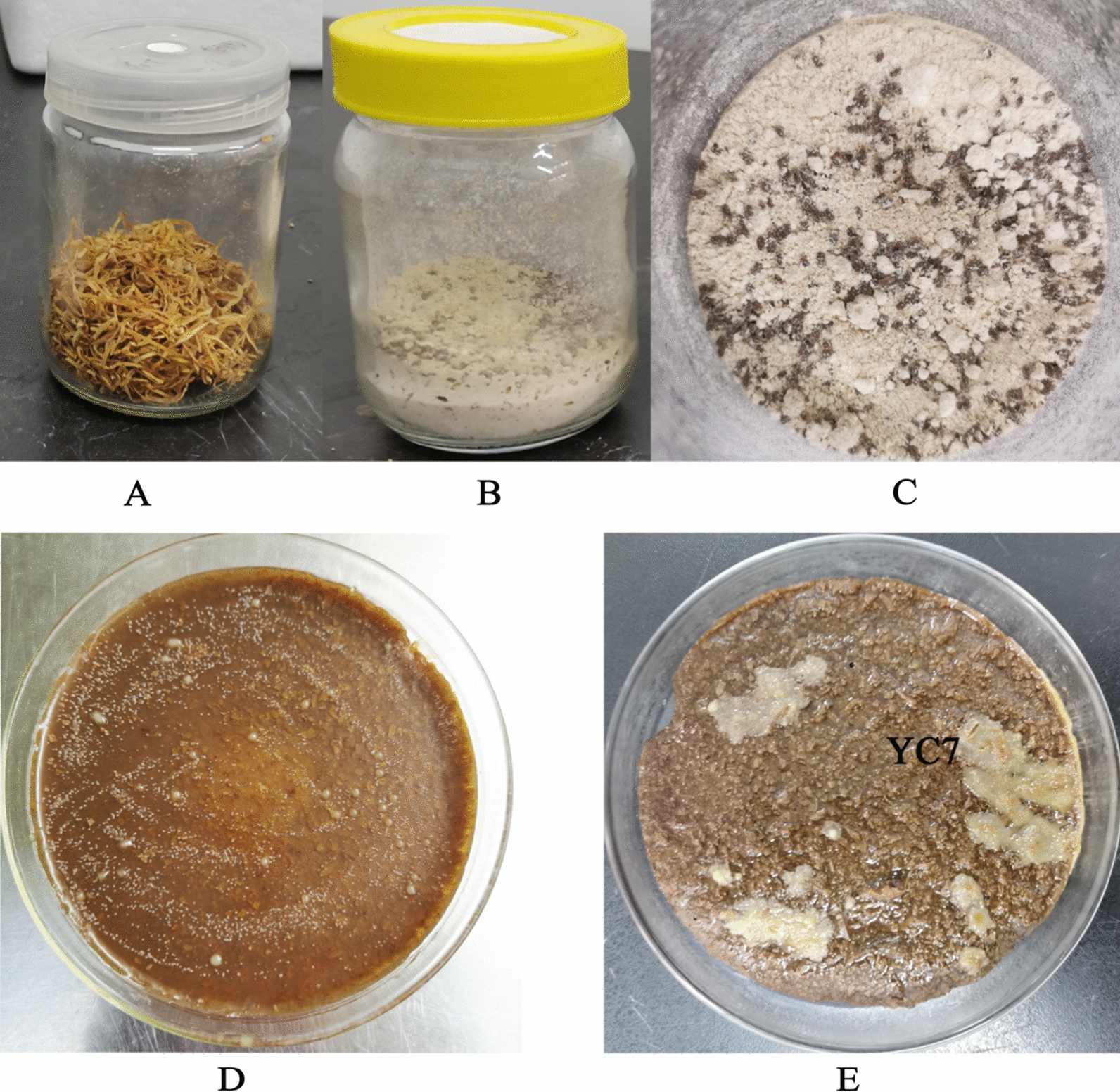
Isolation and screening results of the nicotine degradation bacteria. **A** tobacco leaves where *Lasioderma serricorne* was isolated; **B** *L.serricorne* were grown in a flour medium; **C** *L.serricorne* were reproduced in a flour medium; **D** Screening results on the tobacco medium; E. Rescreening results on the tobacco medium plus nicotine

Tobacco beetles were obtained from contaminated tobacco leaves and then cultured in a flour medium (Fig. 1A–C). After washing three times with 75% ethanol, the beetles were frozen, ground, and spread onto tobacco plates in a gradient dilution. Approximately 50 different single colonies were selected and numbered according to the differences in colony size, morphology, and color on the plates (Fig. 1D). A total of 12 strains of nicotine-degrading bacteria were isolated and screened from the tobacco beetle, and these were named J1, J2, and YC1–YC10 (Table 1).

**Table 1:** Morphological characteristics of high nicotine degradation bacteria

No. of strains	Morphological characteristics
J1	small colony, round, smooth surface, white
J2	small colony, round, smooth surface, white
YC1	small colony, round, smooth surface, light yellow
YC2	big colony, round, slimy surface, yellow
YC3	small colony, round, smooth surface, creamy white
YC4	big colony, round, wrinkled surface, white
YC5	small colony, round, smooth surface, white opaque
YC6	small colony, round, slimy surface, yellow
YC7	big colony, round, smooth surface, milky white
YC8	small colony, round, slimy surface, milky white
YC9	small colony, round, wrinkled surface, light yellow
YC10	big colony, round, wrinkled surface, pale yellow

The tobacco leaves were dried and pulverized in a pulverizer, and 1 g, 2 g, 4 g, 8 g, and 10 g were separately collected and placed into 100 mL of medium to make the different tobacco media with gradually increasing nicotine concentrations used for rescreening the nicotine-degrading bacteria. By comparison, the YC7 strain grew well on the highest tobacco leaf content (10 g/100 mL), and this strain had the maximum degradation capability (Fig. 1E). The strain *Bacillus* sp. YC7 has been deposited in the China Pharmaceutical Culture Collection (CPCC) under the accession number CPCC 101379.

### Taxonomic identification results of nicotine-degrading bacteria

**Fig. 2:**
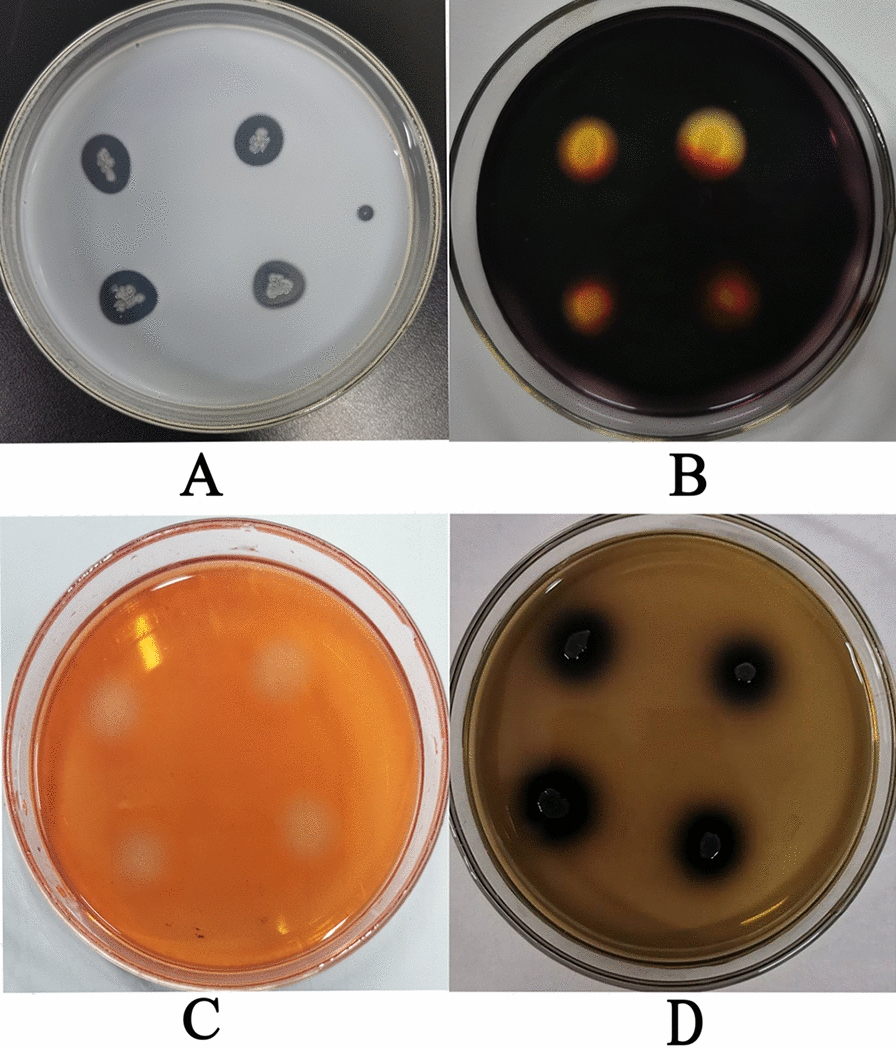
Detection of the YC7 enzyme activity: **A** YC7 protease activity assay; **B** YC7 amylase activity assay; **C**. YC7 Congo red staining; **D** YC7 aescin staining

The results of theβ-glucosidase, cellulase, protease, and amylase activity assay by strain YC7 are shown in Fig. 2. The YC7 strain appeared as transparent circles on milk (Fig. 2A), starch plates (Fig. 2B), and Congo red (Fig. 2C). Moreover, obvious darkened zones were observed on the Aescin plates (Fig. 2D). The results indicated that the YC7 strain produced β-glucosidase, cellulase, protease, and amylase.

**Fig. 3:**
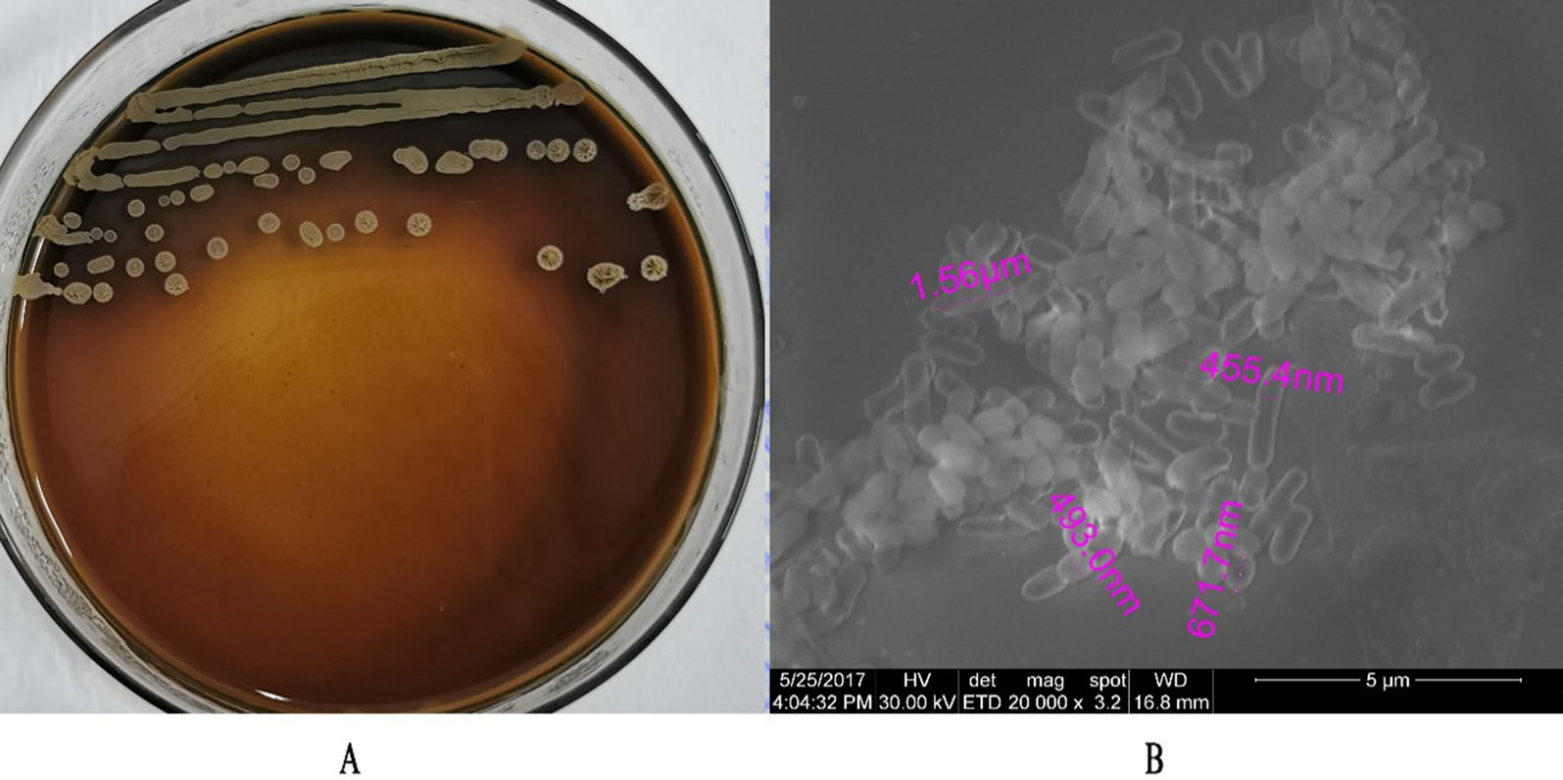
YC7 morphological characteristics: **A** YC7 colony morphology; **B** Cellular morphological characteristics of YC7

The YC7 strain morphology is shown in Fig. 3. The YC7 colonies were smooth, opaque, white, dry, and had uneven edges. The colonies had diameters of 1.5–2.5 mm after incubation for two days at 37 °C on LB plates (Fig. 3A). The YC7 strain was aerobic and gram-positive. Cells were bacilli with blunt rounded ends that were approximately 0.5–1.0 μm wide by 1.3–2.5 μm long and occurred singly or in short chains, and were motile and spore forming (Fig. 3B).

**Fig. 4:**
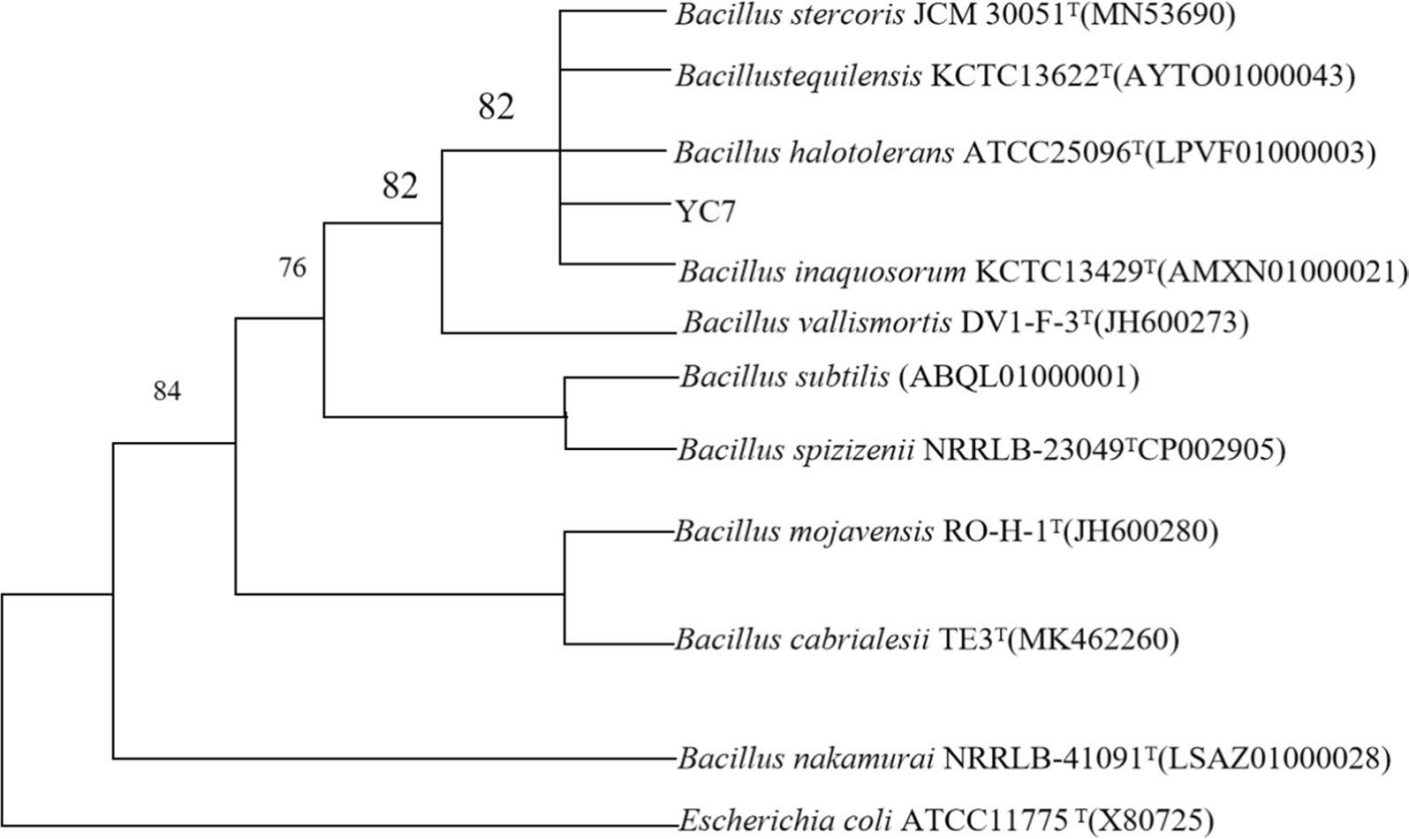
YC7 phylogenetic tree based on the16S rRNA gene sequence

The 16S rRNA gene of the YC7 strain was amplified and obtained. The sequencing results were uploaded into the Ezbiocloud database, and a phylogenetic tree was constructed that showed the YC7 strain had the highest similarity with *B. halotolerans* ATCC 25096^ T^ at 99.73%. The morphologic and phylogenetic analyses results identified the YC7 strain as the *Bacillus* genus (Fig. 4).

### Test results of the YC7 nicotine degradation activity

**Fig. 5:**
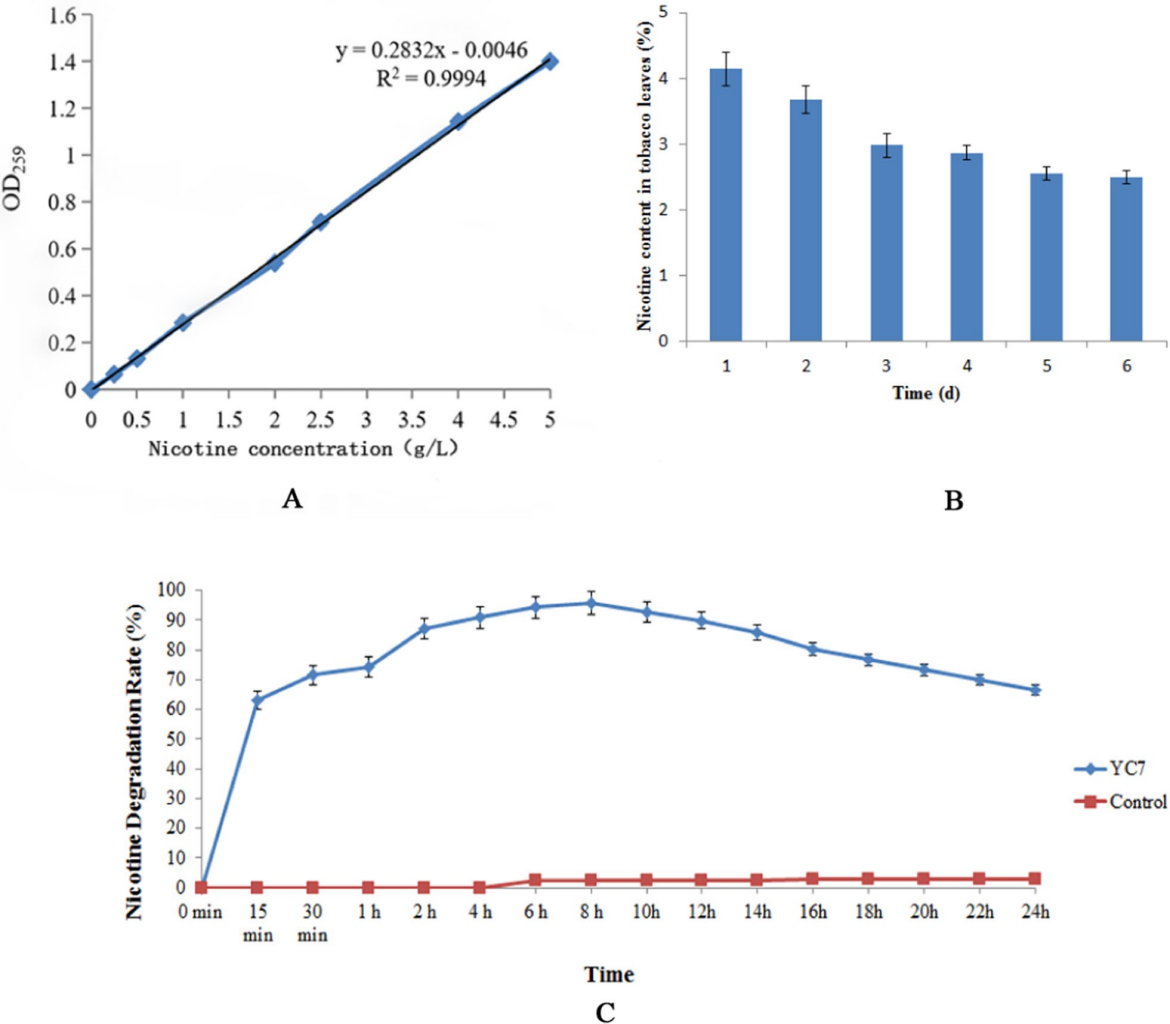
**A** Standard curve of the nicotine concentration detected via UV absorption. **B** Changes in the nicotine content in tobacco leaves treated with the YC7 fermentation solution treatment at different days. **C** YC7 nicotine degradation rates at different incubation times at 37 °C and a 1 g/L nicotine concentration

The nicotine degradation capabilities were determined using the ultra-violet (UV) absorption method. In the 0.25 − 5 g/L nicotine concentration range, the relationship curve between the absorbance value and nicotine concentration was obtained, and the standard curve was Y = 0.2832X − 0.0046 and R^2^ = 0.9994 (Fig. 5A).

The nicotine degradation activity test results in tobacco leaves using the fermentation solution are shown in Fig. 5B. After five days of treatment with the YC7 bacterial solution treatment on tobacco leaves, the nicotine content decreased from 4.15% to 2.55% of the control, and the nicotine degradation rate of the tobacco leaves was 38.55% (Fig. 5B).

The nicotine degradation activity test results in pure nicotine using the fermentation solution of YC7 are shown in Fig. 5C. The nicotine degradation rate of YC7 was 63.16%, 90.90%, and 95.65% at 15 min, 4 h, and 8 h, respectively, at 37 °C and a nicotine concentration of 1 g/L.

### Identification of the protein with degradation activities

**Fig. 6:**
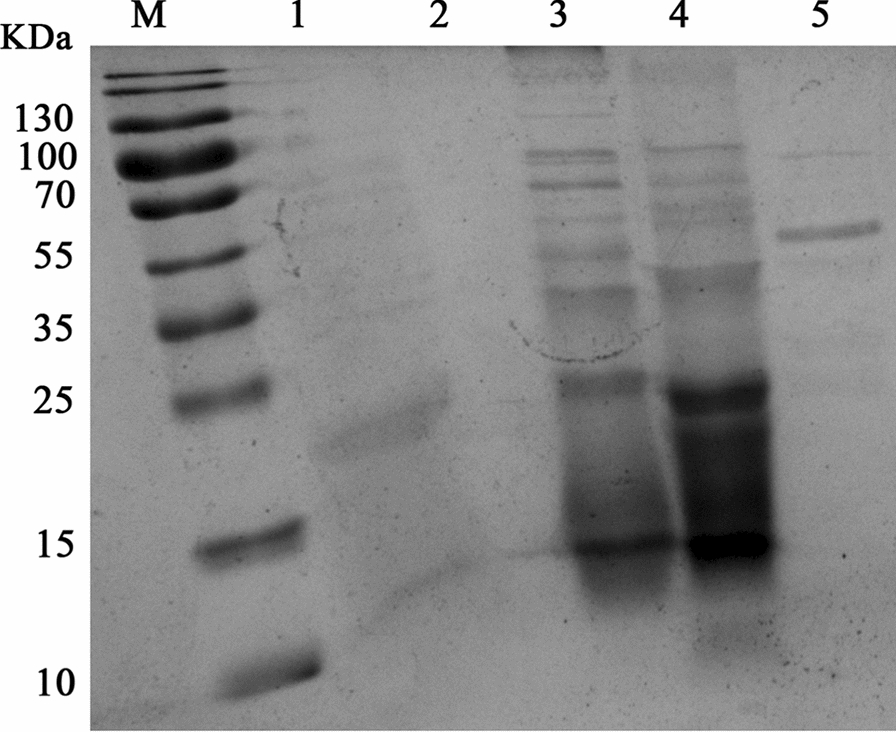
SDS-PAGE of the crude enzyme solutions of YC7. M: Marker; lane 1: fermentation solution; lane 2: liquid culture medium; lanes 3–5 are the ammonium sulfate saturation: 0–30%, 30–50%, and 50–80%, respectively

The protein activities involved in the nicotine degradation function of *Bacillus* sp. YC7 were identified using the bioassay-guided method. The sodium dodecyl sulfate–polyacrylamide (SDS-PAGE) electrophoresis results of the crude enzyme solution precipitated by ammonium sulfate grading are shown in Fig. 6. The maximum degradation capability was in the 50–80% salting out of the ammonium sulfate fraction. The primary protein band with approximately 45 KDa was pooled (Fig. 6 line 5), and its N-terminal amino acid sequence analysis was performed. The N-terminal sequence of the first 10 amino acids was blasted in the National Center for Biotechnology Information (NCBI) GenBank. The result of the N-terminal amino acid sequence indicated that it had 100% similarity to NAD(P)/FAD-dependent oxidoreductase from *B. halotolerans* (WP_202657750) according to BLAST in the NCBI.

### Whole-genome analysis of the YC7 strain

**Fig. 7:**
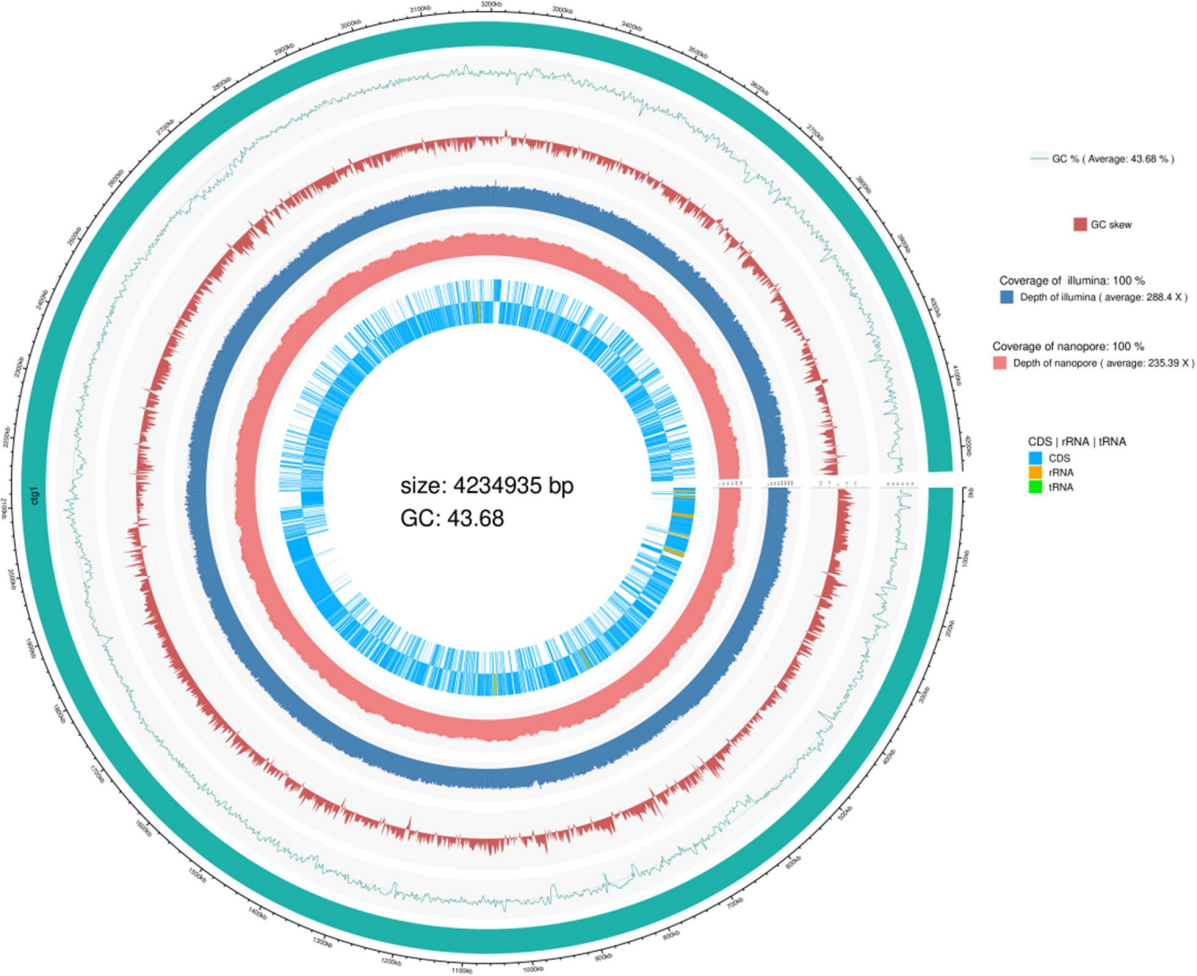
The genome sequence of the YC7 strain

The genome completion map of the YC7 strain is shown in Fig. 7. The whole-genome sequencing (Genbank No.: PRJNA967139) revealed that the genomic sequence length of YC7 was 4,234,935 bp, with a 43.68% guanine cytosine (GC) content and a total of 4444 genes, namely, 4222 coding protein sequence genes (CDS) with an average length of 876 bp, accounting for 87.33% of the whole genome, 87 tRNA genes, 30 rRNA genes, 1 tmRNA gene, and 104 misc-RNA genes.YC7 was functionally annotated in the Pfam, Nr, gene ontology (GO), Kyoto Encyclopedia of Genes and Genomes (KEGG), and clusters of orthogulous genes (COG) pathway general databases. The Pfam classification showed that YC7 had 10 protein families, namely, AAA-tran, AAA-16, AAA-22, MFS-1, AAA-21, AAA-29, SMC-N, DUF258, BPD-transp-1, and AAA. The GO classification showed that the YC7 genes had functions in three areas: biological processes, cellular components, and molecular functions. The COG classification showed that YC7 had 12 class II metabolic pathways in the metabolism category, including the metabolism of terpenoids and polyketides, amino acid metabolism, and the metabolism of other amino acids pathways. The Nr classification showed that the amino acid sequence of the YC7 protein was largely identical to that of *Bacillus*. The KEGG classification showed that YC7 had a large number of genes that perform amino acid transport and metabolism (E: amino acid transport and metabolism) and transcription (K: transcription) functions. In the KEGG pathway, YC7 was found to have NDH (nicotine dehydrogenase) in the pyridine pathway, SPM (3-succinoylpyridine monooxygenase) in the pyrrole pathway, and NFO (N-formylmaleamate dehydrogenase) and maleamate amidohydrolase (AMI) in the pyrrole pathway. The whole-genome sequencing analysis demonstrated the presence of key genes and enzymes for nicotine degradation in YC7 at the genetic level.

### Construction of the knockdown mutant strain and the degradation activity test results

**Fig. 8:**
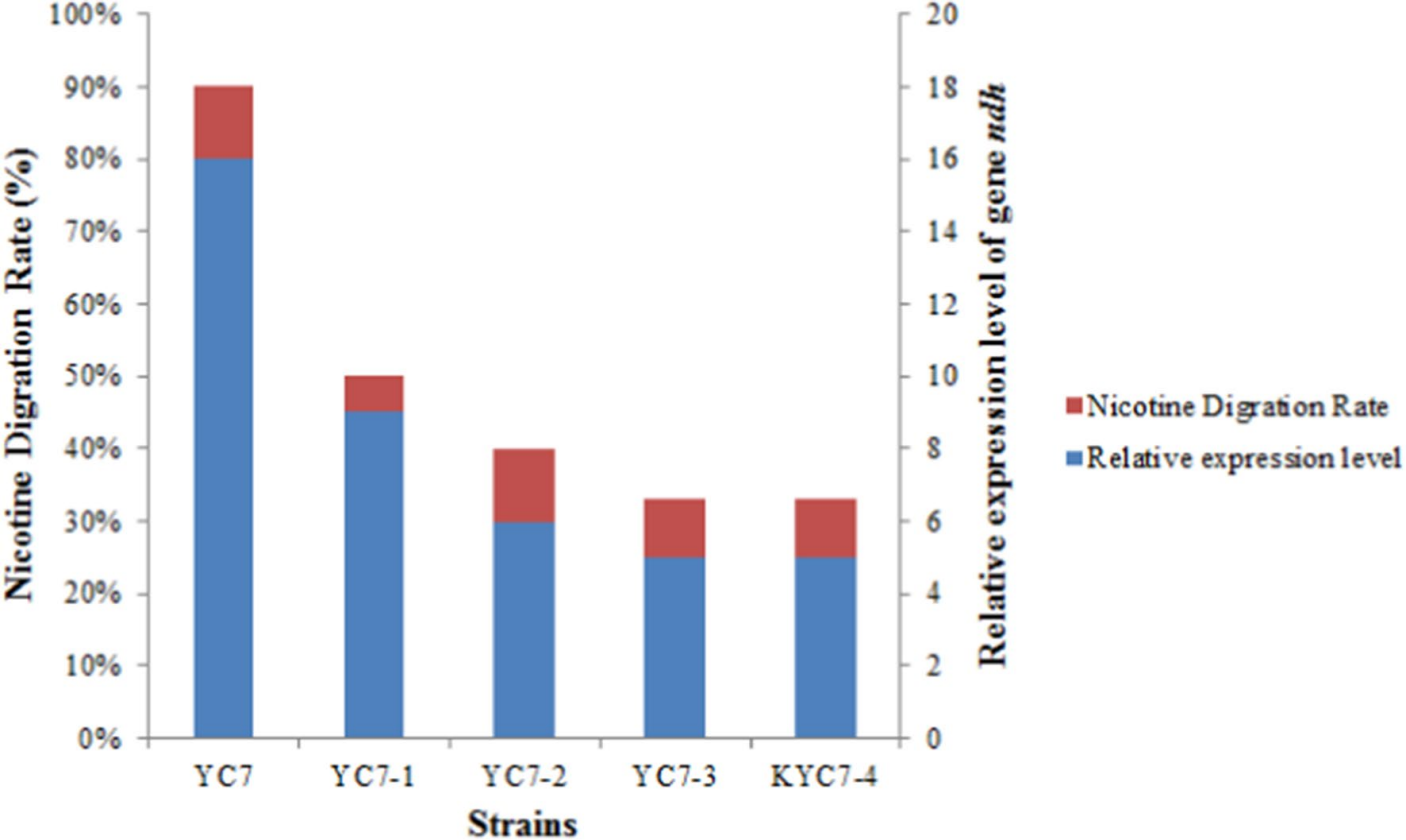
Test results of the nicotine-degradation capabilities and the *ndh* gene expression level of the mutant strains

The sgRNA near the promoter region was inserted into the plasmid pBD1, and the recombinant plasmid was transformed into *B. nematicidae* YC7, resulting in a gene knock-down mutant. The results of qPCR showed that there was no significant change in the expression of the *ndh* gene in the strains expressing sgRNA compared with the wild strain without isopropyl-beta-D-thiogalactopyranoside (IPTG) induction. However, under IPTG induction, the *ndh* gene expression in the knock-down mutant was significantly lower than that in the control strain (Fig. 8). When the IPTG concentration was 0.5 mmol/L, 1.0 mmol/L, and 1.5 mmol/L, the nicotine degradation rate decreased to 40%, 33%, and 33% respectively, which was significantly different from that of the control degradation rate of 90%. The results confirmed the nicotine degradation function of the gene *ndh*.

### Molecular docking and the RT-qPCR functional verification results

The docking energy of nicotine dehydrogenase (NDH) of YC7 with nicotine was –5.51 kcal/mol.

**Fig. 9:**
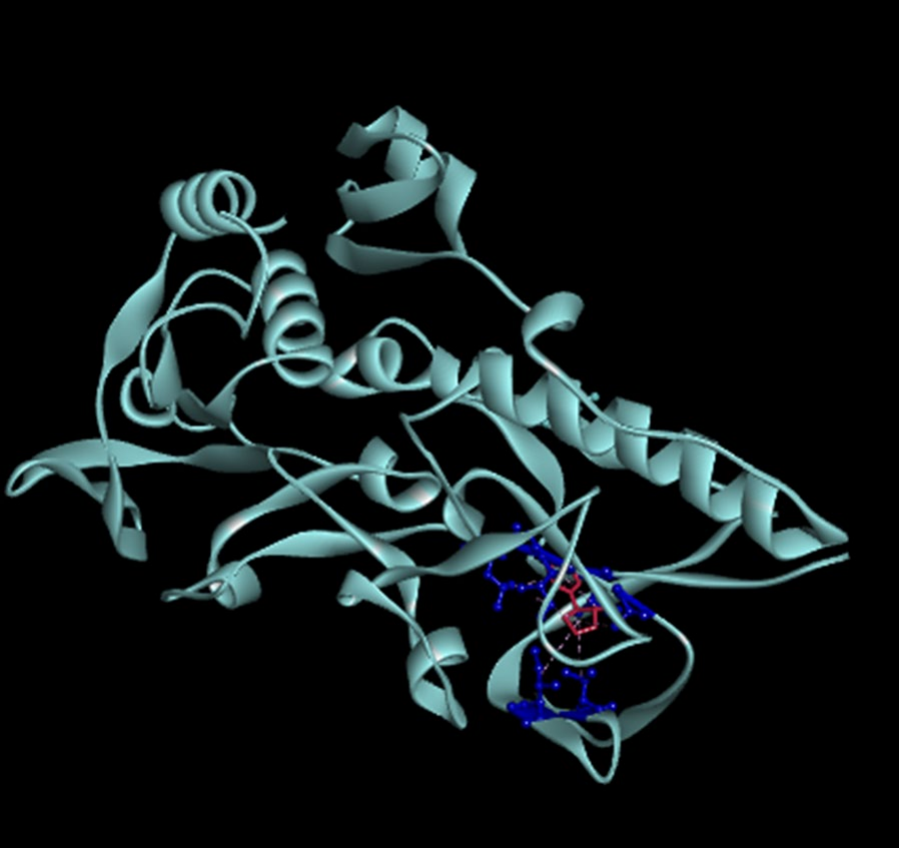
The docking results of the YC7 nicotine dehydrogenase (NDH) with nicotine

The NDH B chain of YC7 at positions 21, 66, 88 Ile (isoleucine), 73 Val (valine), 86 Ala (alanine), 89 Gly (glycine), and 90 Leu (leucine) were able to dock with nicotine (Fig. 9).

The real-time fluorescence quantitative PCR results showed that the relative expression of the *ndh* gene was up-regulated 11.67 times induced by 1 g/L nicotine for 24 h compared with the control group without nicotine induction. When the YC-7 strain was induced by the 0.8% tobacco leaf extract for 24 h, the relative expression of the *ndh* gene was up-regulated 1.37 times compared with the nicotine non-induced control group. The qPCR validation results showed that the expression of the *ndh* gene in YC7 was significantly up-regulated after nicotine induction, and the *ndh* gene played an important role in nicotine degradation.

## Discussion

Tobacco processing generates a significant amount of nicotine-containing waste, posing a considerable threat to both the environment and human health. Consequently, the exploration of nicotine-degrading microorganisms (NDMs) has become a pressing issue (Brandsch 2006; Liu et al. 2015). Among these, the tobacco beetle stands out as a remarkable organism, capable of completing its life cycle by consuming tobacco leaves while effectively metabolizing most of the nicotine into harmless alkaloids. Additionally, the tobacco beetle’s gut microbiota play a crucial role in nicotine degradation.

Over the years, NDMs have garnered increasing attention, with various strains being identified, such as *Arthrobacter nicotinovorans*, *Microsporum gypseum*, *Pellicularia filamentosa* JTS-208, *Nocardioides* sp. strain JS614 (Ganas et al. 2008), *Pseudomonas* sp. CS3 (Wang et al. 2012a), *Shinella* sp. HZN7 (Ma et al. 2014; Qiu et al. 2014, 2016b), *Bacterium* sp. strain J54 (Jiang et al. 2021), and *Pseudomonas* sp. 41 (Liu et al. 2015). Many of these NDMs have been isolated from tobacco plantation soil, tobacco leaves, and tobacco wastes. Furthermore, their metabolic pathways and degradation mechanisms have been extensively studied, providing valuable insights into nicotine degradation processes, including *A. nicotinovorans* (Liu et al. 2015), *Agrobacterium tumefaciens* S33 (Liu et al. 2015), *Aspergillus oryzae* and *P. putida* S16 (Tang et al. 2008), *Pseudomonas* sp. S-1 (Pan et al. 2018), *P. plecoglossicida* TND35 (Raman et al. 2014), *P. geniculata* N1 (Liu et al. 2014), *Ochrobactrum* sp. strain SJY1 (Yu et al. 2015), *Arthrobacter* sp. aRF- 1 (Ruan et al. 2018), *P. putida* (Hu et al. 2019), and *Pseudomonas* spp (Li et al. 2010).

Bacterial nicotine catabolism involves three main pathways: the pyridine pathway, the pyrrolidine pathway, and a variant of the pyridine and pyrrolidine pathways (VPP pathway) (Mu et al. 2020). Notably, certain NDMs, such as *Pseudomonas geniculata* N1, utilize a hybrid pathway of pyridine and pyrrolidine for nicotine degradation (Huang et al. 2020). In-depth studies on the key enzymes involved in these pathways, such as 6-hydroxypseudooxynicotine amine oxidase (HisD) and 6-hydroxypseudonicotinamide oxidase, have shed light on the molecular mechanisms of nicotine degradation (Liu et al. 2020).

Furthermore, *A. tumefaciens* S33 exhibits a unique capability to degrade nicotine through a novel combination of the pyridine and pyrrolidine pathways (Wang et al. 2012b). This intriguing ability enables it to effectively remove nicotine from tobacco waste and convert it into essential functionalized pyridine precursors, with significant applications in the production of valuable drugs and pesticides. A comprehensive genomic analysis of strain S33, along with its transcriptome grown in both glucose-ammonium and nicotine media, has been reported (Huang et al. 2017). Remarkably, the evolution of this hybrid pathway was not simply the fusion of genes from the two pathways but resulted from a complex process of horizontal gene transfer. These insightful studies shed new light on the molecular mechanisms underlying this innovative hybrid pathway of nicotine degradation (Gurusamy and Natarajan 2013).

The crucial role of specific enzymes in the nicotine degradation process has also been extensively explored. For instance, Li et al. identified 6-hydroxy-3-succinyl pyridine hydroxylase as a central step in nicotine degradation catalyzed by* A. tumefaciens* S33 (Li et al. 2014). The NDH enzyme complexed with 6-hydroxypseudooxynicotine oxidase plays a significant role in the mixed nicotine degradation pathway of *A. tumefaciens* S33 (Li et al. 2016). Additionally, periplasmic NDH NdhAB utilizes pseudoazurin as its physiological electron acceptor in *A. tumefaciens* S33 (Yu et al. 2017). Studies by Wang et al. identified 6-hydroxypseudooxynicotine dehydrogenase as a key enzyme in the mixed pyridine and pyrrolidine pathway of nicotine degradation in *A. tumefaciens* S33, with EtfAB serving as a physiological electron acceptor (Wang et al. 2019b). Furthermore, Rid was found to enhance the 6-hydroxypseudooxynicotine dehydrogenase reaction during nicotine degradation in *A. tumefaciens* S33 (Shang et al. 2021b). Shang J. et al. identified a NAD-specific 6-hydroxy-3-succinoyl-semialdehyde-pyridine dehydrogenase from the nicotine-degrading *A.tumefaciens* S33 strain, which exhibits a wide range of substrates with potential applications for enzyme catalysis (Shang et al. 2021a).

The study of nicotine degradation genes and enzymes has yielded significant insights. A novel 31-kb nicotine-degrading gene cluster, *ndp*, in strain TY exhibits a distinct genetic organization compared to the *vpp* cluster found in strains* O. rhizosphaerae* SJY1 and *A. tumefaciens* S33 (Wang et al. 2017). Haixia Wang et al. reported the identification of two novel sets of genes, *ndr*A1A2A3, and *ndr*B1B2B3B4, crucial for nicotine degradation by strain *Sphingomonas melonis* TY (Wang et al. 2016). Additionally, Wang L. et al. studied an *A. nicotinovorans* molybdenum hydroxylase KDH, which plays a signicant role in nicotine degradation (Wang et al. 2021). The *agnH* gene in *A. tumefaciens* strain SCUEC1 and the *ocnE* gene in *O. intermedium* SCUEC4 involved in nicotine-degradation pathways, have also been investigated (Xia et al. 2020; Yu et al. 2021). Notably, the nicotine-degrading enzyme, NicA2, has demonstrated its potential in reducing nicotine levels in the blood, nicotine distribution in the brain, and nicotine discrimination and reinforcement in rats (Pentel et al. 2018). As a result, optimizing the nicotine-degrading enzyme, NicA2, holds promise for potential applications in nicotine addiction treatment (Thisted et al. 2019). Moreover, the first structure of nicotine oxidoreductase (NicA2) was determined using X-ray crystallography. Tararina MA et al. employed crystallography coupled with kinetic analysis to unveil the mechanistic underpinnings of this nicotine-degrading enzyme (Tararina et al. 2016, 2018). The study revealed fast kinetics, highlighting rate-limiting oxidation and the role of the aromatic cage in the mechanism of the nicotine-degrading enzyme NicA2 (Tararina et al. 2021). Furthermore, a computational analysis of the nicotine oxidoreductase mechanism using the our own n-layered Integrated molecular Orbital and Molecular mechanics (ONIOM) method was performed (Yildiz 2021). Additionally, Dulchavsky M et al. reported that cytochrome c acts as the natural electron acceptor for nicotine oxidoreductase (Dulchavsky et al. 2021).

Moreover, genetic manipulation of nicotine-degrading bacteria has shown promising results, with *Pseudomonas* sp., JY-Q being a preferred strain for tobacco waste treatment (Li et al. 2019). Functional modules derived from nicotine-degrading gene clusters have been found to have additive effects, enhancing bacterial degradation efficiency. Differential effects of the homologous transcriptional regulators, NicR2A, NicR2B1, and NicR2B2, and endogenous ectopic strong promoters on nicotine metabolism in *Pseudomonas sp.* strain JY-Q have been found (Huang et al. 2021). In addition, functional enhancement of a metabolic module via endogenous promoter replacement for *Pseudomonas* sp. JY-Q to degrade nicotine in tobacco waste treatment has been accomplished (Li et al. 2021).

The mutagenomic analysis of various strains has unveiled specific genomic structures and regulatory mechanisms governing nicotine degradation. Genome-editing strategies would be well worth investigating to substantially increase the efficiency of bacterial nicotine degradation (Zhang et al. 2022). In addition, crystallographic and kinetic analyses have provided a mechanistic basis of nicotine-degrading enzymes (Tararina et al. 2018).

In this study, 12 nicotine-degrading bacterial strains were isolated and screened, with *Bacillus* sp. YC7 being identified as a key player in the nicotine degradation process. The results of whole-genome sequencing analysis, molecular docking and qPCR validation provided further insights into the interaction of YC7’s NDH enzyme with nicotine, corroborating its significance in nicotine degradation.

The findings from this study have not only introduced a novel concept for nicotine-degrading bacteria screening but have also expanded the repertoire of nicotine-degrading microorganisms, opening up new possibilities for future research. The construction of overexpression-engineered and knockout-engineered strains of NDH offers potential avenues for further validation and exploration of key genes in nicotine degradation metabolic pathways.

In conclusion, this study adds to the growing body of knowledge on nicotine degradation and highlights the potential of NDMs in addressing the pressing issue of nicotine-containing waste. Further investigations and engineering efforts hold promise for improving nicotine degradation efficiency and exploring the diverse applications of these microorganisms in environmental and industrial contexts.

## Data Availability

All of the data and material are available upon request to the corresponding author.
